# Temporal and Permanent Changes Induced by Maxillary Sinus Lifting with Bone Grafts and Maxillary Functional Endoscopic Sinus Surgery in the Voice Characteristics—Systematic Review

**DOI:** 10.3390/dj10030047

**Published:** 2022-03-11

**Authors:** Rafael Delgado-Ruiz, Daniele Botticelli, Georgios Romanos

**Affiliations:** 1 Department of Prosthodontics and Digital Technology, Stony Brook University, Stony Brook, New York, NY 11766, USA; 2 ARDEC Academy, 47923 Rimini, Italy; daniele.botticelli@gmail.com; 3 Department of Periodontology, Stony Brook University, Stony Brook, New York, NY 11766, USA; georgios.romanos@stonybrookmedicine.edu; 4 Department of Oral Surgery and Implant Dentistry, Dental School (Carolinum), Johann Wolfgang Goethe University, 60596 Frankfurt, Germany

**Keywords:** sinus lifting, bone grafts, functional endoscopic sinus surgery, voice changes, post-operative complications

## Abstract

Sinus surgery procedures such as sinus lifting with bone grafting or maxillary functional endoscopy surgery (FESS) can present different complications. The aims of this systematic review are to compile the post-operatory complications of sinus elevation with bone grafting and FESS including voice changes, and to elucidate if those changes are either permanent or temporary. The Preferred Reporting Items for Systematic Reviews and Meta-Analyses (PRISMA) were used, and the literature was exhaustively searched without time restrictions for randomized and non-randomized clinical studies, cohort studies (prospective and retrospective), and clinical case reports with ≥4 cases focused on sinus lift procedures with bone grafts and functional endoscopic maxillary sinus surgery. A total of 435 manuscripts were identified. After reading the abstracts, 101 articles were selected to be read in full. Twenty articles that fulfilled the inclusion criteria were included for analysis. Within the limitations of this systematic review, complications are frequent after sinus lifting with bone grafts and after FEES. Voice parameters are scarcely evaluated after sinus lifting with bone grafts and no voice changes are reported. The voice changes that occur after FESS include a decreased fundamental frequency, increased nasality, and nasalance, all of which are transitory.

## 1. Introduction

The human voice is an air-driven, vibration-produced, and resonance-enhanced phenomenon that requires the concurrent work of practically all of the body [1]. Echternach et al. described the three elements that produce the voice: “air source (lungs, trachea, and larynx), voice/sound source (vocal folds), and the modification system (vocal and nasal tract).” [2] The characteristic features of the voice are defined by changes in those components or their interactions [2].

When the sound moves from the source (vocal folds) along with the other anatomical structures, the fundamental frequency (F0) is changed, acquiring a complex form that can be strengthened or attenuated by the size and shape of the vocal and nasal tracts [3].

The analysis of the individual normal voice characteristics and their changes requires the measurement of at least six sound parameters, including the following: F0, cepstral peak prominence (CPP), jitter, jitta, and shimmer, and noise-to-harmonic ratio (HNR) [4,5].

The F0 is defined as the mean frequency produced by the vibration of the vocal folds [6,7]; the CPP evaluates the periodicity in the frequency and helps in determining the voice pitch or its perturbations [8]; jitter measures the fluctuations of the voice frequency, jitta measures the change in timbre in voice over short periods, and shimmer measures the amplitude of the peak-to-peak frequency during the voice cycle [9]. Finally, the HNR ratio evaluates the periodic and aperiodic components of the speech signal and reflects the airflow efficiency in producing vibration of the vocal folds [10,11].

The interaction of the vocal and nasal tracts influences the character of the sound but also plays an essential role in the production of nasal vowels and nasal consonants as well as the shaping of the voice timbre and its resonance [12]; this is attributed to the anatomical characteristics of both vocal and nasal tracts and the main paired paranasal sinuses (frontal, ethmoidal, maxillary, and sphenoidal) [13]. It seems that the surgical modification of the vocal and nasal tracts and surgery of the paranasal sinuses can result in alterations to the voice characteristics [14].

For example, Viswanath et al. reported that transsphenoidal surgery (a surgical procedure to remove certain pituitary tumors) could result in transient affectation of the voice and speech [15]. In addition, Kim et al. [16] confirmed that the endoscopic transsphenoidal approach resulted in hypernasality (increased nasal resonance) [17], and increased jitter and shimmer [16,17].

Regarding the maxillary sinus, Tepper et al. [18] completed a study on professional singers to determine voice changes after sinus lifting and grafting. Their results showed that none of the evaluated parameters (F0, CPP, sound pressure level, jitter, shimmer, and HNR) changed after the surgical procedures. In contrast, Ungor et al. [19], in a recent study, found voice alterations after maxillary sinus lifting with bone grafting and simultaneous implant insertion in patients that required bilateral maxillary sinus augmentation. Their results showed a reduction in the volume of the sinus spaces and changes in the voice quality demonstrated by altered jitter and jitta, and stated that voice changes after sinus surgery, although not reported, can frequently occur [19].

Regarding maxillary sinus surgery for dental-implant-related procedures, four main approaches are used [20,21,22,23]:

- Sinus lift + bone graft
- Sinus lift + bone graft + implant insertion
- Sinus lift + implant insertion without bone graft
- Sinus endoscopic surgery

In the first three approaches (a, b, and c), the Schneiderian membrane is separated from the lateral wall, floor of the sinus cavity, and the medial wall, and then elevated to create space for the insertion of the graft particles and the implant, which allows the subsequent bone formation [20,21,22,23]. Meanwhile, maxillary functional endoscopic sinus surgery (FESS) (d) is used to treat sinus disease and to restore Ostia patency [24,25], and provides direct view and better control of sinus surgery procedures [24,25,26]. In brief, the minimally invasive access of an endoscope provides a direct vision of the middle turbinate and the middle meatus (osteomeatal complex). Thus, the ostium can be restored, infected mucosa can be removed, and foreign bodies can be localized and extracted [27].

Four main endoscopic approaches provide different access to the maxillary sinus: uncinectomy with middle meatal antrostomy (access the posterior area of the maxillary sinus), mega-antrostomy or modified maxillectomy (access inferior portions of the maxillary sinus), pre-lacrimal recess (wide access to the maxillary sinus), and radical medial maxillectomy (widest access to the maxillary sinus) [28].

In general, in the case of maxillary sinus lift with bone grafts, the post-operatory follow-up comprises evaluating the grafted site, measuring thickening of the sinus membrane, and clinical presence of pain and infection, among others [29,30,31]. Indeed, the release of inflammatory mediators occurs after sinus surgery, resulting in transitory sinusitis with sub-acute characteristics [32,33]; in addition, altered mucociliary function and infection have been reported [34].

In the case of maxillary sinus endoscopic surgery, post-operatory evaluations assess the formation of scar bands around the natural ostium and surgical ostium, the presence of secondary ostial stenosis, the existence of osteitis of the uncinated process, the confirmation of sinus function and ventilation, and evaluation of the damage of the nasolacrimal duct [35].

Although both techniques are well established, the literature is scarce about voice changes and the type of voice alteration that patients can experience after sinus augmentation procedures and FESS of the maxillary sinus. The present systematic review is written to determine the post-operatory complications after sinus elevation with bone grafting, including voice changes, to determine complications and voice changes produced by functional endoscopic sinus surgery (related to the maxillary sinus), and to elucidate if those changes are either permanent or temporary.

## 2. Materials and Methods

This systematic review protocol is registered at the International Prospective Register of Systematic Reviews (PROSPERO) with ID# CRD42022292739. The Preferred Reporting Items for Systematic Reviews and Meta-Analyses (PRISMA) guidelines were also followed to search and compile the information for this systematic review.

A PICOT format including population (male and female adult patients), intervention (maxillary sinus lifting with bone grafting and functional endoscopic maxillary sinus surgery), comparison (healthy patients), outcome (complications including membrane perforation, sinusitis, sinus membrane thickening, infection, inflammation, pain, and voice changes), and time (post-operatory until 1 year) was used to answer the following research questions: “Which are the post-operative complications of sinus floor elevation with bone grafts and functional endoscopic maxillary sinus surgery? Are voice changes reported within the complications of sinus surgery? What types of voice changes are reported, and are the changes transitory or permanent?

To answer those questions, the search was completed in Medline, EMBASE, Google Scholar, and PubMed from October 2021 to January 2022 for literature in the English language.

The following search terms were used: “maxillary sinus lifting AND bone grafting AND complications” OR “maxillary sinus floor elevation AND grafting AND complications” OR “maxillary sinus AND augmentation AND complications” OR “sinus surgery AND voice” OR “maxillary sinus surgery AND voice” OR “functional endoscopic maxillary sinus surgery AND complications” OR “functional endoscopic maxillary sinus surgery AND voice changes” OR “endoscopy maxillary sinus surgery AND voice.” Filters for this type of article were applied to include only clinical studies, randomized and non-randomized studies, cohort studies (prospective and retrospective), and clinical case reports with ≥4 cases.

### 2.1. Selection Criteria

The studies had to be written in the English language, without time limitations regarding the year of publication. It was decided that quantitative data summarizing complications after sinus surgery, including sinus elevation with bone graft and maxillary functional endoscopic surgery, should be included. The included manuscripts were also screened to determine whether voice changes were included within the follow-up evaluations, and if voice changes were reported, then which voice parameters changed, and the duration of the voice changes (temporary or permanent). The following inclusion and exclusion criteria were considered:

#### 2.1.1. Inclusion Criteria

Randomized and non-randomized clinical studies, cohort studies (prospective and retrospective), and clinical case reports with ≥4 cases. Focused on sinus lift procedures with bone grafts and functional endoscopic maxillary sinus surgery.

Listing post-operative complications with or without voice changes after sinus lifting with bone grafts and after functional endoscopic maxillary sinus surgery.

Follow-up period one year or less.

Adult male and female patients at the moment of the intervention within the range of 25 to 80 years of age.

#### 2.1.2. Exclusion Criteria

Articles are written in other languages different than English.

Animal and in-vitro studies.

Repeated or duplicated studies.

Case reports with <4 cases.

Child or teenager patients.

Sinus lifts without bone graft.

Sinus lift including simultaneous implant insertion.

Cancer or tumor-related patients.

Sinus surgical procedures not related to those of the inclusion criteria.

#### 2.1.3. Definition of Variables

##### Sinus Lifting with Bone Grafts

The procedure involves access to the maxillary sinus using a lateral window or a transcrestal approach (with rotary or ultrasonic instruments), followed by separation of the maxillary sinus membrane (sinus elevation or sinus lifting) and the insertion of a bone graft or bone substitute. It may include or not the use of membranes. Must consider the previously described inclusion and exclusion criteria.

##### Functional Endoscopic Surgery (FESS) of the Maxillary Sinus

The procedure uses an endoscope to directly visualize the middle turbinate and the middle meatus to restore Ostia patency and treat sinus disease, including only uncinectomy with middle meatal antrostomy and pre-lacrimal recess access.

##### Voice Temporal Change

A transitory change in any of the following voice parameters: fundamental frequency (F0), cepstral peak prominence (CPP), jitter, jitta, and shimmer, and noise-to-harmonic ratio (HNR) produced after the surgical intervention. The parameter returns to baseline values within the first year.

##### Voice Permanent Change

A permanent change in any of the following voice parameters: fundamental frequency (F0), cepstral peak prominence (CPP), jitter, jitta, and shimmer, and noise-to-harmonic ratio (HNR) produced after the surgical intervention. The parameter does not return to baseline values within the first year.

### 2.2. Evaluators’ Calibration

Data forms containing the inclusion and exclusion criteria (checklists) were created. The evaluators reviewed the inclusion and exclusion criteria and graded the provided definitions for the criteria. The evaluators’ answers could assign two possible values: 0 = incorrect, 1 = correct. The correct points for each evaluator and the closest values between evaluators indicated the intra- and inter-evaluator agreement.

### 2.3. Article Selection

An initial search was completed within the available literature for clinical studies, randomized and non-randomized studies, cohort studies (prospective and retrospective), and clinical case reports with ≥4 cases with titles relevant to the research question (R.D). The abstracts were read in full to confirm that the articles satisfied the inclusion criteria (R.D and G.R). In case of disagreement between investigators, a third investigator (D.B) decided to exclude the article in dispute. The eligible articles were included in the review and data extraction. An additional manual search was completed within to confirm that there were not duplicated studies.

### 2.4. Data Collection

Qualitative and quantitative data were collected, including the following: type of sinus floor elevation procedure, the number of patients, follow-up time, complication after sinus elevation with bone grafts (i.e., perforations, sinusitis, thickening of the sinus membrane, infection, inflammation, hemorrhage, dehiscence, and pain among others), complications of functional endoscopic maxillary sinus surgery (listed by the authors of the included articles) including voice changes as well as the time until the complication was resolved, and other findings. If voice changes were evaluated, the type of voice change and time that the voice change remained until recovery. The resulting data were organized in tables in chronologic order of appearance (oldest to newest).

### 2.5. Risk of Bias

We followed the recommendations by Ma et al. 2020 [36], which stated that a specific risk of bias scale should be used for each type of study. For non-randomized studies, the MINORS scale [37]; for randomized controlled trials, the risk of bias tool (RoB 2) [38]; for prospective and retrospective studies, the CASP checklist [39]; and for case reports, the Joanna Briggs Institute critical appraisal (JBI checklist) were used [40].

### 2.6. Data Analysis

Quantitative synthesis of the data will be completed if comparable studies are available. Preferably, a random-effects meta-analysis will be implemented. A narrative data synthesis providing descriptive statistics of the evaluated variables will be provided if the data are heterogeneous.

## 3. Results

The initial search returned 453 manuscripts. After reading the titles, 352 articles were excluded because they were mid-term or long-term studies, duplicated studies, included implants or implant loading. Then, the abstracts of the remaining 101 articles were read, and 20 articles were removed because they included dental implants simultaneously with the sinus floor elevation and grafting. Afterward, the full texts of the remaining 81 articles were read in total and based on the exclusion and inclusion criteria, 20 articles were included for analysis.

**Figure 1:**
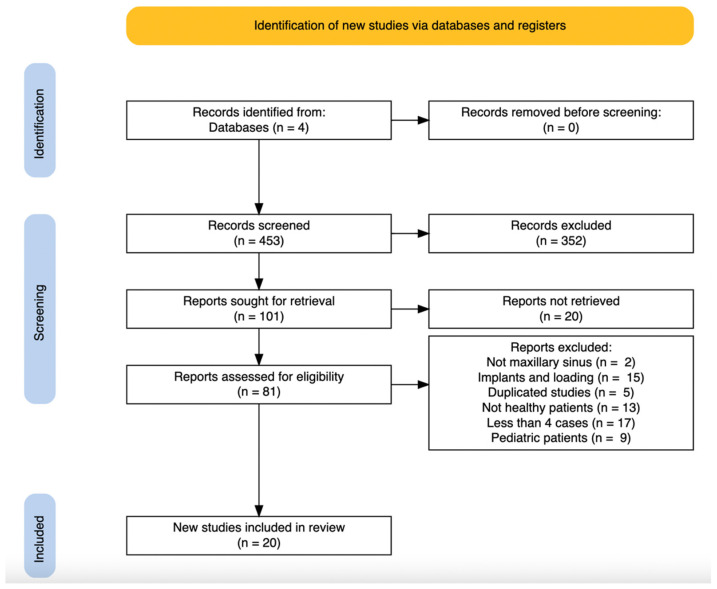
Scheme illustrating the search strategy. From the 453 initially identified manuscripts and following the inclusion and exclusion criteria, 20 manuscripts were finally included for this review.

The PRISMA2020: R package and ShinyApp for producing PRISMA-2020-compliant flow diagrams by Zenodo [41] were used for the generation of the work-flow diagram (Figure 1) of the twenty included articles; fifteen were related to complications after sinus elevation with bone grafts [18,42,43,44,45,46,47,48,49,50,51,52,53,54,55], and five were related to complications after FESS of the maxillary sinus [56,57,58,59,60]. Given the heterogeneity of the included studies, a meta-analysis was not possible; therefore, only descriptive data is provided.

### 3.1. Sinus Elevation with Bone Graft

In the group of sinus lift with bone grafts, five randomized controlled trials (33.33%) [47,48,51,52,54], four cohort studies (26.66%) [49,50,53,55], and six case studies (40%) were included [18,42−46].

In total, 646 patients were subjected to 930 sinus lift procedures with bone grafts. The most frequent complications of sinus lift with bone grafts were membrane perforations (101 sinuses or 10.86%), followed by sinusitis (13 sinuses or 1.39%) and bleeding/hematomas (12 sinuses or 1.29%), wound dehiscence (9 sinuses or 0.96%), and inflammation (4 sinuses or 0.430%).

From the 20 studies included, 19 studies used a lateral window approach; only 1 study used a transcrenstal approach. The post-operative complications were all reported for lateral window approaches. The complications were reported between day 0 and 6 months (Table 1).

Voice changes were rarely evaluated in sinus lifting with bone grafting studies. Only one sinus lifting with bone grafting study (6.66% of 15 studies) evaluated voice changes [18], and no changes in the evaluated voice parameters were found.

### 3.2. Functional Endoscopic Sinus Surgery

In the FESS group, three cohort studies (60%) [57,58,59] and two case studies (40%) were included [56,60]. The studies reported a total of 325 patients, in which 210 FESSs were completed. The complications registered after FESS included post-nasal drip and other eye symptoms (11 sinuses or 5.23%), cheek pain and tenderness (3 sinuses or 1.42%), blocked middle meatal antrostomy (3 sinuses or 1.42%), adhesions (3 sinuses or 1.42%), relapse of infection (2 sinuses or 0.95%), only epiphora (2 sinuses or 0.95%), remnants of the uncinate process (2 sinuses or 0.95%), nasal hemorrhage (1 sinus or 0.47%), nasal obstruction (1 sinus or 0.47%), and nasal discharge (1 sinus or 0.47%).

Voice changes were evaluated in two studies in the FESS group (40% of 5 studies) [58,60]. Regarding the type and duration of the voice changes, it was found that the fundamental frequency decreased until the third month [58], and nasalance and nasality increased until the first year of follow-up [60] (Table 2).

### 3.3. Risk of Bias Assessment for the Included Studies on Sinus Lifting with Bone Grafting

**Figure 2:**
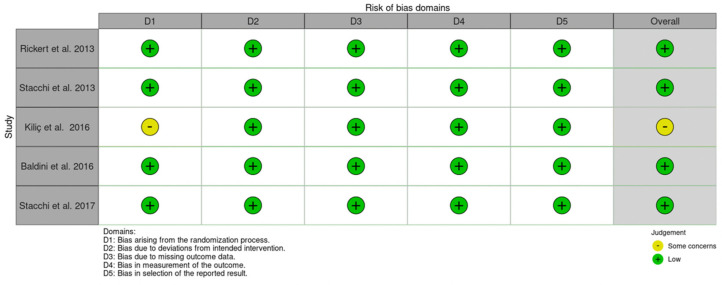
Risk of bias for the randomized control studies using the RoB-2 tool. There was one study with some concerns from the randomization process.

Five randomized controlled trials were included for risk of bias assessment using the ROB-2 tool [38]. Four studies showed a low risk of bias, and one study showed a moderate risk of bias. The moderate risk of bias originated from concerns from the randomization process (lacked a precise description) (Figure 2).

Six case studies were included and evaluated with the Joanna Briggs Institute (JBI) critical appraisal tool for case series studies [40]. From the ten evaluated items, it was observed that the condition was measured in a standardized manner, and valid methods were used to identify the condition in 86.6% of the studies. Meanwhile, the follow-up outcomes and statistical analysis were lacking in 86.6% of the studies. The other items presented variable results (Table 3).

Four cohort studies (three prospective, one retrospective) were included for grading using the Critical Appraisal Skills Program checklist (CASP) [39]. All the studies addressed the issue, measured the exposure and the outcomes appropriately, established proper follow-up protocols, and their results fitted within the available evidence. The confounding factors were not identified or considered in the analysis of the results in any of the studies (Table 4).

### 3.4. Risk of Bias Assessment for the Included Studies on FESS

Two case studies were included and evaluated with the Joanna Briggs Institute (JBI) critical appraisal tool for case series studies [40]. From the ten items evaluated in the appraisal, it was observed that the condition was measured in a standardized manner, the participants were appropriately included, the demographics and outcomes were all reported, and appropriate statistics were completed (100% of the studies). Meanwhile, the inclusion criteria, the methods for identifying the condition, and clear reports of the clinical situation were not consistent (50% of the studies) (Table 5).

Three cohort studies were graded using the Critical Appraisal Skills Program checklist (CASP) [39]. All the included studies addressed the focused issue, accurately measured the exposure and outcomes, provided adequate follow-ups, presented the result in detail, and precisely, the results can be applied to the local population, fit with currently available evidence, and presented the clinical implications of their findings. However, in none of the studies were confounding factors considered (Table 6).

## 4. Discussion

This systematic review aimed to determine the post-operative complications after sinus floor elevation with bone grafting, including voice changes, to demonstrate complications and voice changes produced by maxillary FESS, and to elucidate if those changes were either permanent or temporary. It was also aimed to answer the following questions: Are voice changes reported within the complications of sinus surgery? Moreover, what types of voice changes were reported?

The results of this systematic review showed that post-operative complications exist after sinus lifting with bone grafts [42,43,44,46,47,50,51,52,53,54,55], and after FESS [56,57,59]. However, voice change/analysis was rarely included in both techniques’ pre- and post-operative evaluations [18,58,60]. Decreased fundamental frequency (F0) and increased nasalance and voice nasality were observed after FESS [56,57,59].

The risk of bias analysis completed in the present review considered the diversity and heterogeneity among studies and applied specific assessment tools. Thus, randomized clinical trials were assessed using the RoB-2 [38], the cohort studies and case studies were assessed with the CASP checklist, [39] and the case studies were appraised using the JIB tool [40]. This allowed the inclusion and evaluation of more studies relevant to the literature search.

This is the first systematic review that compiles the complications from sinus lift with bone grafts and the complications of maxillary FESS. The present review found that the most common complications were membrane perforations, followed by sinusitis maxillaris, bleeding/hematomas, wound dehiscence, and inflammation. Our results agree with the systematic review by Stacchi et al. [61], who evaluated the intraoperative complications of sinus floor elevation and found that sinus membrane perforation was the most frequent intraoperative complication when the lateral window approach was used to access the sinus cavity.

In addition, our results agree with a systematic review by Ghasemi et al. [62], who evaluated the intra-and postoperative complications of sinus lifting in smokers. They found the same complications plus oroantral fistula and stated that smoking seems to be associated with an increased risk of infection and wound dehiscence.

In the present study, infections were not reported. However, membrane perforations and sinusitis maxillaris were found. As per Schlund et al. [63], both findings can be related to graft infection, which can produce increased morbidity, graft loss, and impaired implant outcomes.

The complications reported in this work from FESS procedures were epiphora (excess tearing), post-nasal drip, eye symptoms, nasal hemorrhage, relapse, infection, adhesions, and nasal obstruction). Similar findings were reported in the systematic review by Bitner et al. [64], who evaluated the outcomes of FESS with or without rhinoplasty. The authors found that the complications were present in both approaches, but the combination of FESS with other surgical procedures may increase the number of complications.

Beyond the complications of FESS reported in the present work, endoscopic sinus surgery possesses other complications depending on the type of endoscopic surgery. For example, for endoscopic middle meatus antrostomy, the natural ostium can be missed, and scarring, injury to the nasolacrimal duct, orbital penetration, and facial numbness can occur. In the case of endoscopy with a balloon, the submucosal passage of the balloon, orbital penetration, pain, facial swelling, and dental numbness might appear [65].

Regarding the evaluation of voice changes after sinus lifting with bone grafting, no changes were observed in the study by Tepper et al. [18]. Four patients received bilateral sinus grafting without measurable consequences on voice parameters in their study. In contrast, the study by Ungor et al. [19] included a larger sample size (17 patients with bilateral sinus lift and immediate implant insertion) and found evident and measurable voice changes in voice professionals. It could be hypothesized that a more traumatic procedure, with a larger portion of the sinus membrane displaced, with significantly reduced sinus volume space (by the grafting material and the implants), increases the impairment of the mucociliary function, producing transitory sinusitis and subsequent voice changes [66,67].

Why did FESSs produce voice changes and sinus lifting whilst bone grafts did not? It seems that the widening of the ostium dimensions produced by FESS not only removes obstructions and restores mucociliary function but also improves the air flow within the nasal passage [68]. This was explained by computational analysis that showed that the aerodynamics of the nasal and sinus cavities changed under inflammatory conditions [69]. In addition, the voice parameters are more frequently evaluated in FESS-related procedures compared to sinus lift with bone grafts.

Meanwhile, the evaluation of the voice characteristics is not a standard procedure before or after the sinus elevation and bone grafts, which can result in a lack of data and overlooking a post-operative phenomenon with subclinical occurrence. It can be hypothesized that the sinus lift and bone grafts (considered an extra-sinusal approach if no perforation occurs) does not change the ostia patency; thus, no voice change can be expected. However, the presence of an accessory ostia blocked by an excess of graft particles may produce changes in the aerodynamics of the osteomeatal clearance. Another aspect that can influence the presence of voice changes is related to the size of perforation and the membrane biotype (thin or thick), which can produce different inflammatory responses [70].

This systematic review possesses some limitations. First, the heterogeneity of the included studies impedes the completion of a meta-analysis; second, the limited number of randomized clinical studies also limits the strength of the summarized evidence; third, the search criteria excluded studies with sinus lifting, grafting, and simultaneous implant placement or sinus lifting without grafting. However, only sinus lifting and bone grafts and FESS were selected to reduce the number of variables that could obscure the possible explanations to the post-operatory complications or possible voice changes. Thus, other factors such as accidental or spontaneous implant displacement into the sinus space, implant infection, or sinusitis related to perforation of the sinus membrane produced by the implant body were excluded [71].

The strengths of the present study are the use of strict and precise inclusion criteria, the use of specific evaluation tools for each group of included studies, and the presentation of a valuable summary of complications after sinus lifting with bone grafts and FESS of the maxillary sinus.

It is imperative to consider the inclusion of voice parameter evaluation after sinus lifting with bone grafts and FESS to understand whether or not voice changes occur, to improve clinical practices, and to prevent unnoticed complications.

## 5. Conclusions

Within the limitations of this systematic review, complications are frequent after sinus lifting with bone grafts and after FEES. Voice parameters are scarcely evaluated after sinus lifting with bone grafts and no voice changes are reported. The voice changes that occur after FESS are decreased fundamental frequency, increased nasality, and nasalance, all of which are transitory.

## Figures and Tables

**Table 1:** Complications after sinus elevation with sinus grafting including reports of voice changes.

Authors/Year/ Type of Study	Intervention	Number of Patients/SINUSES	Follow-Up	Complications								Voice Evaluation	Parameters	Type of Voice Change	Duration
				Membrane Perforation	Sinusitis	Membrane Thickening	Infection	Inflammation	Pain	Hematoma/Bleeding	Wound Dehiscence				
Tepper et al. [18] 2003 Cases study	Bilateral sinus lifting with lateral window. Iliac crest grafts + anorganic bovine bone + platelet rich plasma (PRP)	4/8	NA	-	-	-	-	-	-	-	-	Yes	Singing and speaking voice profile, periodicity, and spectral analysis	No	NA
Shlomi et al. [42] 2004 Comparative cases study	Unilateral and bilateral lateral window + demineralized freeze-dried human lamellar bone sheet + 50/50 mix of autogenous + anorganic bovine bone	36/73	4 to 6 months	20	-	-	-	-	-	-	-	No	NA	NA	NA
Barone et al. [43] 2005 Comparative cases study	Bilateral sinus lifting with lateral window. 100% autogenous bone OR Mix of 50% autogenous + 50% porcine	18/36	5 months	3	-	-	-	-	-	-	-	No	NA	NA	NA
Barone et al. [44] 2008 Comparative cases study	Bilateral sinus lifting the windows were completed either with with rotative instruments (control) piezosurgery (test) + corticocancellous porcine bone	13/26	5 to 6 months	4 (test group) 3 (control group)	-	-	-	-	-	-	-	No	NA	NA	NA
Ucer C. [45] 2009 Cases study	Lateral window + ipsilateral nasal suction + internal collagen membrane + anorganic bovine bone	24/31	NA	-	-	-	-	-	-	-	-	No	NA	NA	NA
Taschieri et al. [46] 2012 Cases study	Lateral window + PRFG clot + deproteinized bovine bone combined with the liquid fraction of PRFG	8/8	NA	2	1	-	-	-	1	3 hematomas	-	No	NA	NA	NA
Rickert et al. [47] 2013 Randomized controlled clinical trial	Bilateral sinus lifting with rotative instruments (control) and piezosurgery (test) + Lateral window + autogenous grafts particles and blocks	36/72	1,2,3 and 12 weeks	4 (test group) 4 (control group)	-	-	-	-	-	-	-	No	NA	NA	NA
Stacchi et al. [48] 2013 Randomized controlled clinical trial	Unilateral sinus lifting with lateral window or lateral erosion + bone grafts (xenograft OR allograft)	72/72	Day 0	4 (lateral window)	-	-	-	-	-	-	-	No	NA	NA	NA
Lie et al. [49] 2015 Prospective study	Bilateral sinus lifting With lateral window + Mix of autogenous bone and xenograft OR resorbable membrane made of poly (D,L)- lactide (PDLLA)	5/10	Up to 6 months	-	-	-	-	-	-	-	-	No	NA	NA	NA
Schwarz et al. [50] 2015 Retrospective study	Sinus lifting with lateral window + mix of autologous bone and deproteinized bovine bone	300/407	NA	34	11	-	-	-	-	-	5	No	NA	NA	NA
Kiliç et al. [51] 2016 Randomized clinical trial	Sinus lifting with lateral window + beta-tricalcium phosphate OR beta-tricalcium phosphate + platelet rich plasma	18/18	10 days to 6 months	3	-	-	-	-	-	-	-	No	NA	NA	NA
Baldini et al. [52] 2016 Randomized clinical trial	Bilateral sinus lifting with large (control) OR small (test) window + deproteinized bovine bone	16/32	7, 14, 30, and 180 days	4 (control group) 3 (test group)	-	-	-	-	-	3	-	No	NA	NA	NA
Alayan et al. [53] 2017 Prospective controlled	Unilateral or bilateral sinus lift with lateral window + mix of autogenous bone with anorganic bovine bone (control) OR anorganic bovine bone with collagen (test)	60/60	1 week 2 weeks and 5 months	8 (test group)	1 (control)	-	-	3 (control) 1 (test)		4 hematoma (control) 1 hematoma (test)	3 (control) 1 (test)	No	NA	NA	NA
Stacchi et al. [54] 2017 Randomized controlled	Bilateral sinus lifting with lateral window prepared with piezosurgery (control) OR bone scrapers (test) + Hydroxyapatite particles	25/50	Day 0	3 (control group) 4 (test group)	-	-	-	-	-	1minor hemorrhage (test)	-	No	Na	NA	NA
Lopez-Quiles et al. [55] 2018 Prospective non-controlled	Transcrestal + Osteotome + balloon lifting + anorganic bovine bone	27/27	5–24 to months	1	-	-	-	-	-	-	-	No	NA	NA	NA

**Table 2:** Complications reported after maxillary functional endoscopy sinus surgery. The functional endoscopic surgery consisted in all the cases with partial uncinectomy, middle meatal antrostomy, and enlargement of the maxillary sinus ostium. FESS: functional endoscopic sinus surgery; NA: not available.

Authors/Year/Type of Study	Intervention	Number of Patients/Sinuses	Follow-Up	Complications	Voice Evaluation	Parameters	Type of Voice Change	Duration
Penttila et al. [56] 1995 Comparative cases study	Functional endoscopy (FESS) Versus Caldwell-Luc (CL) for treating chronic maxillary sinusitis	143 patients in total 71 patients Caldwell-Luc unilateral 72 patients Functional endoscopy unilateral	Up to 1 year	Score of 35 in CL Score of 2 in FESS Cheek pain tenderness 54 in CL and 3 in FESS Epiphora 2 in CL and 2 in FESS Other post-nasal drip, eye symptoms, cosmetic dryness 10 in CL and 11 in FESS	No	NA	NA	NA
Chiapasco et al. [57] 2009 Retrospective study	Functional endoscopy sinus surgery for removal of displaced implants into the maxillary sinus (without oro-antral communication) OR Anterior-lateral window for the removal of displaced implants into the maxillary sinus (in case of oro-antral communication OR Functional endoscopy + lateral window in case or obstruction of the maxillary ostium and oro-antral communication	6 patients/6 sinus 17 patients/17 sinus unilateral 4 patients/4 sinus unilateral	1, 6 and 12 months	1 nasal hemorrhage in a FESS case 1 case of relapse and infection	No	NA	NA	NA
Hernández-García et al. [58] 2020 Prospective study	Functional endoscopy sinus surgery ant their effects on voice and speech recognition In healthy patients, with nasal polyps and undergoing sinus surgery	53 patients 26 FESS 27 healthy	Baseline 2 weeks and 3 months	No postsurgical complications were described	Yes	Grade, Roughness, Breathiness, Asthenia, and Strain (GRBAS assessment)	FESS produces decrease of F0 (fundamental frequency) Change in the vocal tract that increased the error of recognition in FESS patients	3 months
Yadav S et al. [59] 2021 Prospective controlled study	Functional endoscopy sinus surgery patients with chronic maxillary sinusitis Comparing the standard technique uncinectomy + middle meatus antrostomy (MMA) Versus swing door technique	60 patients 30 with the standard technique 30 with the swing door technique	2 and 6 weeks	At 2 weeks, 8 complications were observed in patients treated with the standard technique as follows: 2 Remnants of uncinate process 3 Blocked MMA 3 Adhesions At 6 weeks only 1 minor complication was found in a patient treated with the standard technique In addition, the following symptoms were observed at 6 weeks (more symptoms in the standard method than the swing door technique: Nasal obstruction Postnasal drip Nasal discharge	No	No	No	NA
Yang et al. [60] 2021 Comparative cases study	Unilateral functional endoscopy sinus surgery in patients with chronic rhinosinusitis.	42 patients 21 with limited surgery (1 sinus) 21 with wide opening surgery (more than 1 sinus ipsilateral)	Before surgery 6 months after surgery 12 months after surgery	Not reported	Yes	Objective nasality outcomes measured with a nasometer AND Subjectively nasality assessed by a Visual Analogue Scale (VAS) by the patients, and by questionnaires by their partners	Increased nasalance and nasality	Objective nasalance score increased 1 year after FESS Subjective self-reported nasality assessment improved significantly postoperatively.

**Table 3:** Case series for sinus lifting with bone grafts evaluated with the Joanna Briggs Institute (JBI) critical appraisal tool for case series studies [40]. Ten items were evaluated per included article. The checklist determines four possible outcomes for each item: if the item was evaluated (Yes), if the item was not evaluated (No), the item is not clear (unclear), and the item does not apply (not applicable). For interpretation purposes, the higher the number of “No” answers, the lower the quality of the study. This appraisal tool was used with permission from the Joanna Briggs Institute.

CASE SERIES	1. Clear Inclusion Criteria			2. Condition Measured in a Standard, Way			3. Valid Methods for Identification of the Condition			4. Consecutive Inclusion of Participants?			5. Complete Inclusion of Participants?			6. Clear Report of Demographics			7. Clear Report of Clinical Information			8. Outcomes or Follow-Up Clearly Reported?			9. Clear Reporting of Sites’/Clinics’ Demographic Information?			10. Appropriate Statistics		
**Tepper et al. 2003**	Unclear			Yes			No			No			Yes			Yes			No			No			Yes			No		
**Shlomi et al. 2004**	No			Yes			Yes			Unclear			Unclear			Yes			No			No			No			No		
**Barone et al. 2005**	Yes			Yes			Yes			No			Yes			Yes			Yes			No			No			Yes		
**Barone et al. 2008**	Yes			Yes			Yes			No			Unclear			No			Yes			No			No			No		
**Ucer** **2009**	No			No			Yes			Yes			No			No			No			No			Yes			No		
**Taschieri et al. 2012**	Yes			Yes			Yes			No			Unclear			Yes			No			Yes			Yes			No		
RESULTS PERCENTAGES	Y	N	U	Y	N	U	Y	N	U	Y	N	U	Y	N	U	Y	N	U	Y	N	U	Y	N	U	Y	N	U	Y	N	U
	50	33.3	16.6	83.3	16.6	-	83.3	16.6	-	66.6	16.6	16.6	16.6	33.3	50	66.6	33.3	-	66.6	33.3	-	16.6	83.3	-	50	50	-	16.6	83.3	-

**Table 4:** Critical Appraisal Skills Program checklist (CASP). Each one of the items was appraised for each included article and only one of three possible answers was selected based in the definitions (Y = Yes, CT = can’t tell, or N = no). This CASP checklist is licensed by a Creative Commons Attribution-ShareAlike 4.0 International License [39].

Cohort Studies	Section A: Are the Results of the Study Valid?								Section B: What Are the Results?			Section C: Will The Results Help Locally?		
	1. The Study Addresses a Clearly Focused Issue?	2. Was the Cohort Recruited in an Acceptable Way?	3. Was the *Exposure* Accurately Measured to Minimize Bias?	4. Was the *Outcome* Accurately Measured to Minimize Bias?	5a. Have the Authors Identified All Important Confounding Factors?	5b. Confounding Factors in the Design and/or Analysis Were Taken into Consideration	6a. Was the Follow-Up of the Subjects *Complete* Enough?	6b. Was the Follow-Up of Subjects *Long* Enough?	7. What Are the Results of the Study	8. How Precise Are the Results?	9. Do You Believe the Results?	10. Can the Results Be Applied to the Local Population?	11. Do the Results of the Study Fit with Other Available Evidence?	12. What Are the Implications of the Study for Practice?
**Lie et al. 2015** **Prospective study**	Y	N	Y	Y	N	N	Y	CT	CT	N	CT	N	Y	CT
**Schwarz et al. 2015** **Retrospective study**	Y	Y	Y	Y	CT	Y	Y	Y	Y	Y	Y	CT	Y	Y
**Alayan et al.** **2017** **Prospective controlled**	Y	Y	Y	Y	CT	N	Y	Y	Y	Y	Y	Y	Y	Y
**Lopez-Quiles** **et al.** **2018** **Prospective no controlled**	Y	Y	Y	Y	N	N	Y	Y	Y	Y	Y	CT	Y	Y

**Table 5:** Case series for functional endoscopic maxillary sinus surgery evaluated with the Joanna Briggs Institute (JBI) critical appraisal tool for case series studies [40]. Ten items were evaluated per included article. The checklist determines four possible outcomes for each item: if the item was evaluated (Yes), if the item was not evaluated (No), the item is not clear (unclear), and the item does not apply (not applicable). For interpretation purposes, the higher the number of “No” answers, the lower the quality of the study. This appraisal tool was used with permission from the Joanna Briggs Institute.

CASE SERIES	1. Clear Inclusion Criteria			2. Condition Measured in a Standard, Way			3. Valid Methods for Identification of the Condition			4. Consecutive Inclusion of Participants?			5. Complete Inclusion of Participants?			6. Clear Report of Demographics			7. Clear Report of Clinical Information			8. Outcomes or Follow-Up Clearly Reported?			9. Clear Reporting of Sites’/Clinics’ Demographic Information?			10. Appropriate Statistics		
**Penttila et al.** **1995**	No			Yes			No			No			Yes			Yes			No			Yes			Yes			Yes		
**Yang et al.** **2021**	Yes			Yes			Yes			Yes			Yes			Yes			Yes			Yes			Yes			Yes		
RESULTS PERCENTAGES	Y	N	U	Y	N	U	Y	N	U	Y	N	U	Y	N	U	Y	N	U	Y	N	U	Y	N	U	Y	N	U	Y	N	U
	50	50	-	100	-	-	50	50	-	50	50	-	100	-	-	100	-	-	50	50	-	100	-	-	100	-	-	100	-	-

**Table 6:** Critical Appraisal Skills Program checklist (CASP) for the FESS included studies. Each one of the items was appraised and only one of three possible answers was selected based in the definitions (Y = Yes, CT = can’t tell, or N = no). This CASP checklist is licensed by a Creative Commons Attribution-ShareAlike 4.0 International License [39].

Cohort Studies	Section A: Are the Results of the Study Valid?								Section B: What Are the Results?			Section C: Will the Results Help Locally?		
	1. The Study Addresses a Clearly Focused Issue?	2. Was the Cohort Recruited in an Acceptable way?	3. Was the *Exposure* Accurately Measured to Minimize Bias?	4. Was the *Outcome* Accurately Measured to Minimize Bias?	5a. Have the Authors Identified All Important Confounding Factors?	5b. Confounding Factors in the Design and/or Analysis Were Taken into Consideration	6a. Was the Follow-Up of the Subjects *Complete* Enough?	6b. Was the Follow-Up of Subjects *Long* Enough?	7. What Are the Results of the Study	8. How Precise Are the Results?	9. Do You Believe the Results?	10. Can the Results Be Applied to the Local Population?	11. Do the Results of the Study Fit with Other Available Evidence?	12. What Are the Implications of the Study for Practice?
**Chiapasco et al. 2009**	Y	CT	Y	Y	N	N	Y	Y	Y	Y	Y	Y	Y	Y
**Hernández-García et al.** **2020**	Y	Y	Y	Y	N	N	Y	CT	Y	Y	Y	Y	Y	Y
**Yang et al.** **2021**	Y	CT	Y	Y	Y	N	Y	CT	Y	Y	Y	Y	Y	Y

## Data Availability

The data from the articles included in this review are available upon request to the corresponding author.
